# Assessment of beneficial effects and identification of host adaptation-associated genes of *Ligilactobacillus salivarius* isolated from badgers

**DOI:** 10.1186/s12864-023-09623-8

**Published:** 2023-09-07

**Authors:** Yu Wang, Xiaomeng Xu, Huan Chen, Fang Yang, Bo Xu, Kun Wang, Qianwen Liu, Guixin Liang, Ruiqi Zhang, Xin’an Jiao, Yunzeng Zhang

**Affiliations:** 1 Jiangsu Co‐Innovation Center for Prevention and Control of Important Animal Infectious Diseases and Zoonoses, Yangzhou University, Yangzhou, 225009 China; 2 Jiangsu Key Laboratory of Zoonosis, Yangzhou University, Yangzhou, 225009 China; 3 Joint International Research Laboratory of Agriculture and Agri‐product Safety of the Ministry of Education, Yangzhou University, Yangzhou, 225009 China; 4 Key Laboratory of Prevention and Control of Biological Hazard Factors (Animal Origin) for Agrifood Safety and Quality, Ministry of Agriculture of China, Yangzhou University, Yangzhou, 225009 China

**Keywords:** *Ligilactobacillus salivarius*, Badger, Host adaptation, Beneficial effects, Complete genome

## Abstract

**Background:**

*Ligilactobacillus salivarius* has been frequently isolated from the gut microbiota of humans and domesticated animals and has been studied as a candidate probiotic. Badger (*Meles meles*) is known as a “generalist” species that consumes complex foods and exhibits tolerance and resistance to certain pathogens, which can be partly attributed to the beneficial microbes such as *L. salivarius* in the gut microbiota. However, our understanding of the beneficial traits and genomic features of badger-originated *L. salivarius* remains elusive.

**Results:**

In this study, nine *L. salivarius* strains were isolated from wild badgers' feces, one of which exhibited good probiotic properties. Complete genomes of the nine *L. salivarius* strains were generated, and comparative genomic analysis was performed with the publicly available complete genomes of *L. salivarius* obtained from humans and domesticated animals. The strains originating from badgers harbored a larger genome, a higher number of protein-coding sequences, and functionally annotated genes than those originating from humans and chickens. The pan-genome phylogenetic tree demonstrated that the strains originating from badgers formed a separate clade, and totally 412 gene families (12.6% of the total gene families in the pan-genome) were identified as genes gained by the last common ancestor of the badger group. The badger group harbored significantly more gene families responsible for the degradation of complex carbohydrate substrates and production of polysaccharides than strains from other hosts; many of these were acquired by gene gain events.

**Conclusions:**

A candidate probiotic and nine *L. salivarius* complete genomes were obtained from the badgers’ gut microbiome, and several beneficial genes were identified to be specifically present in the badger-originated strains that were gained in the evolution. Our study provides novel insights into the adaptation of *L. salivarius* to the intestinal habitat of wild badgers and provides valuable strain and genome resources for the development of *L. salivarius* as a probiotic.

**Supplementary Information:**

The online version contains supplementary material available at 10.1186/s12864-023-09623-8.

## Background

Animals in the wild usually exhibit specific adaptation traits, such as the safe consumption of pathogen-infected, poisonous foods and tolerance and resistance to various diseases and microbial pathogens [1, 2]; these traits can be partly attributed to the presence of adequate numbers of beneficial microbes in the gut microbiota [3]. For example, many intestinal lactic acid bacteria (LAB) are found to exert beneficial effects on the host in numerous ways and are recognized as an important source of probiotics [4, 5]. Badger (*Meles meles*) is an omnivorous animal species widely distributed in Eurasia [6]. Badgers are found to have a complex feeding habit and consume many types of plant-derived foods, such as wheat, persimmon, and hawthorn fruits, as well as animal-derived foods, such as annelids, mollusks, amphibians, and reptiles [7]. In addition to being a sylvatic repository of zoonotic infectious diseases [6], it is a vital reservoir of health-beneficial probiotics. Stedman et al. isolated and identified multiple LAB strains from badger feces, and identified 40 LAB strains that exhibited significant antimicrobial activities against *Mycobacterium smegmatis*, a species that serves as an indicator for screening antagonistic bacteria against pathogenic *Mycobacterium* species [8]. They then assessed the therapeutic potential of these LAB strains through in vitro immunobiological assessments and comparative genomic analyses and identified several *Lactobacillus* and *Pediococcus* strains that could act as probiotics against infectious diseases in wild animals through various mechanisms, such as modulation of proinflammatory phagocytic responses associated with protection from pathogens [9].

Among LAB species, *Ligilactobacillus* (*L.*) *salivarius* is a widely distributed species that has been identified in a wide range of niches, including the oral cavity, the intestinal tract of humans and several domesticated animals such as pigs and chickens, and fermented foods [10–14]. Many members affiliated with *L. salivarius* isolated from humans are demonstrated to exhibit several beneficial properties, such as inhibiting the growth and reproduction of pathogenic bacteria [5, 15, 16], alleviating inflammatory responses [17], reducing pathogen adhesion to host cells [18], improving the absorption of calcium in the intestinal tract [19], and prolonging the lifespan of the host [20]. Furthermore, multiple *L. salivarius*-affiliated members are found to exhibit good capability to adapt to the challenging intestinal environments, e.g., resistance to acid and bile stresses, which is the fundamental prerequisite for probiotics to exert their beneficial effects [21]. However, majority of the available animal-origin *L. salivarius* strains are from domesticated animals, such as pigs and chickens [22], while data on strains originating from wild animals are lacking.

Comparative genomics is a powerful approach for identifying modified, acquired, or lost genetic features and understanding how they facilitate the evolution and adaptation of strains to specific environmental niches within the same species [23]. Comparative genomic analysis of *L. salivarius* strains mainly isolated from swine, chickens, and humans identified several host-specific genes, which may help *L. salivarius* in adapting to its hosts [22, 24]. However, to the best of our knowledge, no wild animal-originated *L. salivarius* genome has been studied so far. Given that badgers have adapted to consume complex foods including diverse fruits and animals, and harbor zoonotic pathogens in the gut microbiome but do not exhibit disease symptoms [6, 7], the *L. salivarius* strains colonizing the intestinal tract of badgers may harbor specific genomic features that facilitate host adaptation such as the digestion of complex plant materials, and thus isolation of *L. salivarius* strains and exploring their genomic contents can probably benefit the *L. salivarius*-based probiotics development process such as screening of strains with high capability of nutrient conversion and pathogen inhibition and directed strain improvement. Furthermore, most bacterial genome sequencing projects so far have employed short-read-based sequencing technology; the genome sequences assembled from short reads usually cannot unambiguously resolve repeat sequences that may play important biological roles, such as conferring antimicrobial resistance [25, 26], and may lack some accessory genes, core genes, and plasmids [27, 28]. A combination of long- and short-read sequencing technologies can facilitate the assembly process and generate complete and highly accurate genome sequences [29]. In this study, the complete genome sequences of nine *L. salivarius* strains isolated from the feces of badgers were determined, and comparative genomic analysis was performed using 15 complete genomes of *L. salivarius* available in the NCBI database. The analysis focused on the identification of properties contributing to the adaptation to hosts, particularly badgers. This study not only assessed and enriched the strain and genome reservoirs of *L. salivarius* but also provided valuable insights into the host specificity and evolution of *L. salivarius* in different hosts.

## Results

### Some* L. salivarius* strains originating from badgers exhibited beneficial properties

**Fig. 1:**
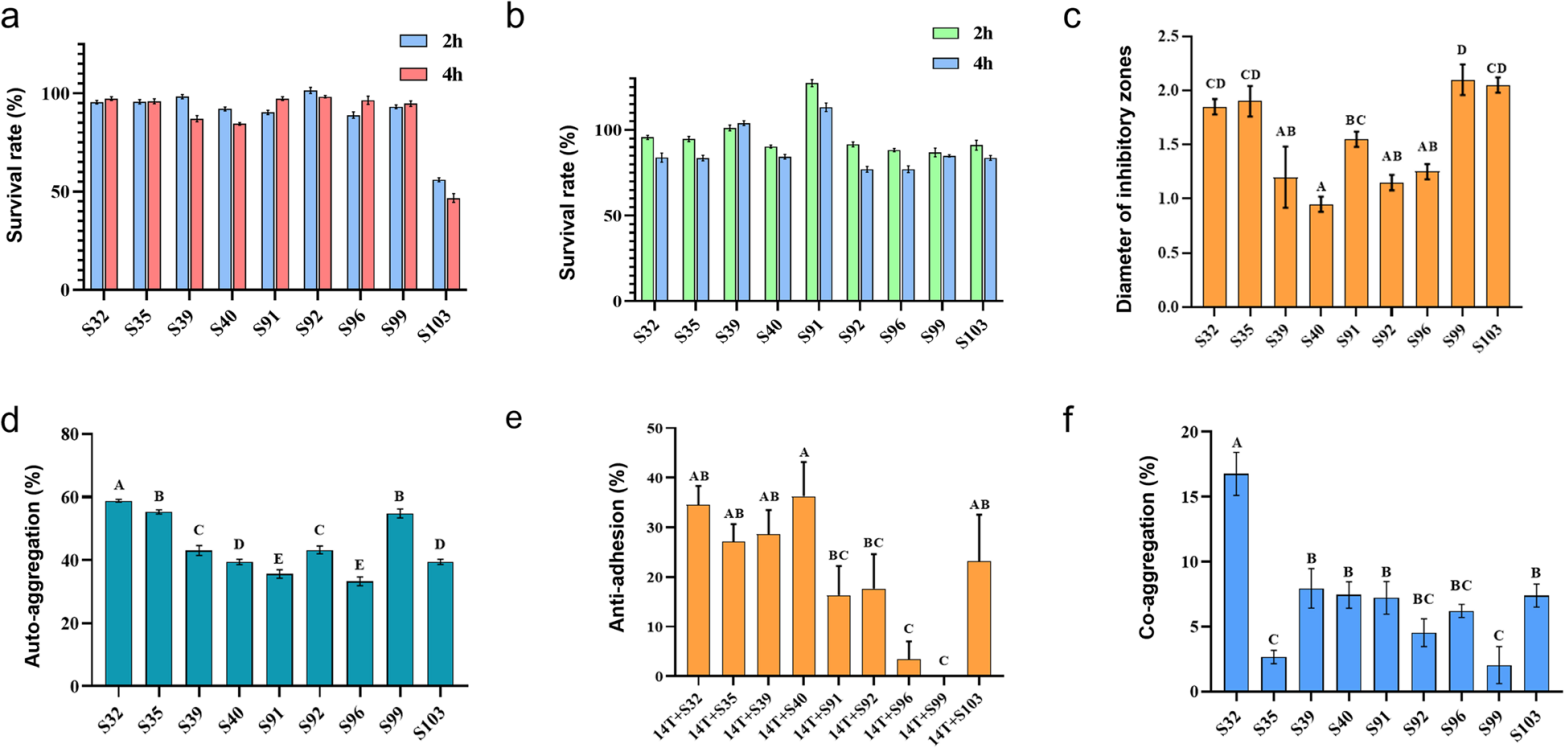
The probiotic property of the nine *L. salivarius* isolates. **a-f** Acid tolerance, bile tolerance, anti-*Salmonella* activity, auto-aggregation abilities, anti-adhesion abilities against *S.* Derby 14T, and coaggregation abilities of nine *L. salivarius* isolates, respectively. Note: Data shown are mean SD of triplicate values of independent experiments. The compact letter display indicates significant differences in pairwise comparisons, and strains with different letters are significantly different (*P* < 0.05, one-way ANOVA, Tukey post hoc test) (**c**-**f**)

Probiotics, such as *L. salivarius*, have to overcome acidic pH and bile acid stresses in the gastrointestinal environments after entering the mouth before they can colonize the intestinal tract [30]. The nine *L. salivarius* strains in our study exhibited high tolerance to acid stress. Eight of these strains showed a > 96% survival rate at pH 3 even after 4 h of challenge (Fig. 1a), which is the regular period for which food stays in the stomach [31]. The *L. salivarius* strains also exhibited good tolerance to 0.15% ox gall (Fig. 1b).

The anti-*Salmonella* effects of the *L. salivarius* strains were then assessed using *S.* Derby 14T as an indicator strain. All nine *L. salivarius* strains exhibited antagonistic activity against *S.* Derby 14T, with strains S32, S35, S99, and S103 showing significantly higher inhibitory activities compared with the other five strains (*P* < 0.05, one-way ANOVA, Tukey post hoc test) (Fig. 1c). LAB can form a barrier that hinders the colonization and infection of pathogens in the intestine through auto-aggregation [32]. Strain S32 exhibited the highest auto-aggregation activity (58.84%), while S35 and S99 ranked to be the second higher group (55.29% and 54.79% for S35 and S99, respectively) (*P* < 0.05, one-way ANOVA, Tukey post hoc test) (Fig. 1d). Successful adhesion to intestinal epithelial cells is a key step for *Salmonella* species to colonize the intestine and cause further infection [33]. Therefore, controlling the adherence of *Salmonella* to intestinal epithelial cells is crucial for preventing and managing *Salmonella* infections. The anti-adhesion ability of the nine *L. salivarius* strains against the adhesion of *S.* Derby 14T to intestinal epithelial cells was assessed in this study. A significant difference was observed in the anti-adhesion ability of the nine *L. salivarius* strains, with strains S40 and S32 reducing the adhesion of *S.* Derby 14T by 36.29% and 33.53%, respectively, and strains S96 (2.35%) and S99 (0%) exhibiting no obvious anti-adhesion effect (Fig. 1e). Moreover, coaggregation of *L. salivarius* strains with pathogens can avoid the colonization of intestinal pathogens by preventing pathogens from attaching to epithelial cells. All nine *L. salivarius* strains exhibited certain coaggregation activities with *S.* Derby 14T, with S32 exhibiting the highest coaggregation capacity of 15.76%, which was significantly higher than that of the remaining eight strains (*P* < 0.05, one-way ANOVA, Tukey post hoc test), while S35 and S99 showing the lowest coaggregation capacity of 3.12% and 2.84%, respectively (Fig. 1f).

### General genomic features of *L. salivarius* strains

**Fig. 2:**
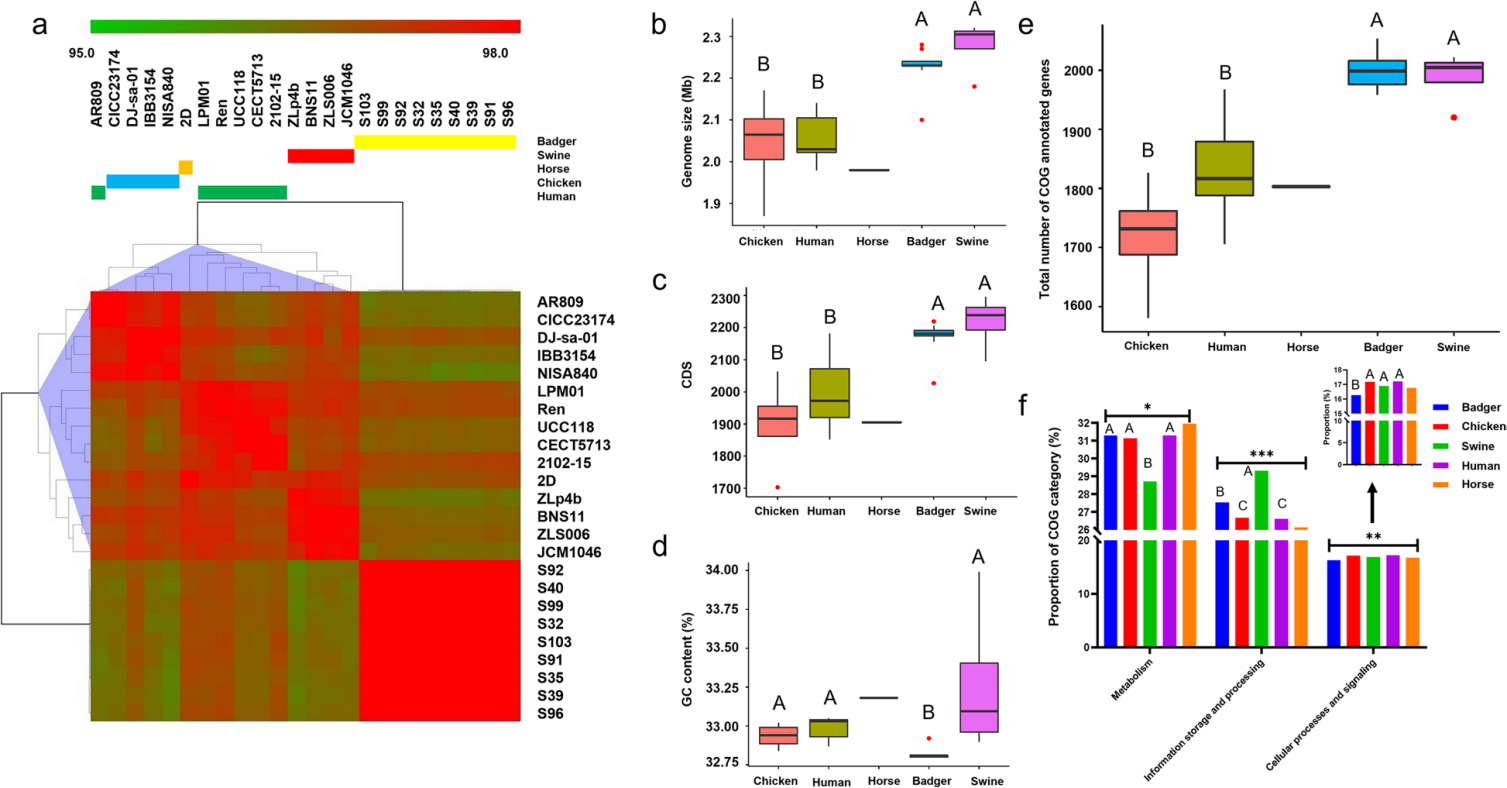
The genomic characteristics of 24 *L. salivarius* strains from different hosts. **a** Heatmap of ANI values based on the sequences of 24 *L. salivarius* strains. **b-f** The comparison of genome size, CDSs, GC content, COG-annotated genes, and COG categories of *L. salivarius* strains from different hosts, respectively. Kruskal–Wallis test with Mann–Whitney post hoc test was used for comparisons (**b-f**). The compact letter display indicates significant differences in pairwise comparisons, and groups with different letters are significantly different (*P* < 0.05). The horse group which contains only one strain was excluded from the statistical anaysis. *, *P* < 0.05; **; *P* < 0.01; ***, *P* < 0.001 as revealed by Kruskal–Wallis test (**f**)

Complete genomes of the nine *L. salivarius* strains were generated using long and short reads. The nine strains harbored one chromosome and one to five plasmids. The genome length was 2.10–2.28 Mb, the GC content was 32.80%–32.92%, and 2027–2219 CDSs were present (Table 1). Comparative genomic analyses were performed using the nine generated badger-originated *L. salivarius* genomes and the 15 complete genomes available in the NCBI (as of March 10^th^, 2022) (Table 1) of *L. salivarius* strains isolated from humans (six strains), swine (four strains), chickens (four strains), and horses (one strain) in order to identify the genomic features specifically present in the strains originating from badgers or in other groups. The ANI values across the 24 strains were higher than 95%, which is recognized as the species boundary, indicating that the strains were affiliated with the same species. Notably, the strains from the same host exhibited much higher ANI values than those from other hosts, reflecting the host specificity of the *L. salivarius* strains (Fig. 2a). Furthermore, the genome size (Kruskal–Wallis test, *P* = 0.001, the horse group that only contained one strain was excluded from the statistical analysis, same herein) (Fig. 2b), number of CDSs (Kruskal–Wallis test, *P* = 0.004) (Fig. 2c), and GC content (Kruskal–Wallis test, *P* = 0.003) (Fig. 2d) significantly differed among the strain groups from different hosts. In particular, the genome size and number of CDSs in the strains originating from swine and badgers were significantly higher than those in the strains originating from chickens and humans (Mann–Whitney test, all *P* < 0.05). Moreover, the strains originating from badgers and swine harbored a significantly higher number of COG-annotated genes compared with those from chickens and humans (Kruskal–Wallis test with Mann–Whitney post hoc test, all *P* < 0.05) (Fig. 2e). Of the COG-annotated genes, the proportion of genes belonging to the COG categories “metabolism” (Kruskal–Wallis test, *P* = 0.02), “information storage and processing” (Kruskal–Wallis test, *P* = 0.002), and “cellular processes and signaling” (Kruskal–Wallis test, *P* = 0.008) significantly differed among the strains from different hosts (Fig. 2f). Specifically, the swine-originated strains harbored fewer metabolism-related genes, and higher information storage and processing-related ones compared with the remaining animal hosts; the badger-originated strains harbored significantly fewer cellular processes and signaling related genes compared with those from humans and chickens (Mann–Whitney test, *P* < 0.05 for both comparisons).

**Table 1:** Summary of *L. salivarius* complete genomes obtained by sequencing and NCBI

Strain	Genome size (Mb)	GC Content	CDS	Host	NCBI accession
S32	2.27	32.81%	2206	Badger	CP114503-CP114508
S35	2.23	32.81%	2177	Badger	CP114509-CP114511
S39	2.23	32.80%	2174	Badger	CP114512-CP114514
S40	2.23	32.80%	2191	Badger	CP114515-CP114517
S91	2.24	32.81%	2181	Badger	CP114518-CP114521
S92	2.28	32.80%	2219	Badger	CP114543-CP114548
S96	2.24	32.81%	2182	Badger	CP114522-CP114525
S99	2.10	32.92%	2027	Badger	CP114501-CP114502
S103	2.22	32.82%	2157	Badger	CP114526-CP114529
CICC23174	2.08	32.84%	1919	Chicken	GCF_001723525.1
DJ-sa-01	1.87	32.98%	1703	Chicken	GCF_003316955.1
IBB3154	2.17	32.90%	2063	Chicken	GCF_011045395.1
NIAS8	2.05	33.02%	1915	Chicken	GCF_000215465.1
2102–15	2.02	33.05%	1983	Human	GCF_021432185.1
AR809	2.03	32.87%	1853	Human	GCF_020535185.1
LPM01	2.03	32.90%	1961	Human	GCF_900094615.1
CECT5713	2.14	33.02%	2181	Human	GCF_000143435.1
Ren	1.98	33.04%	1907	Human	GCF_001011095.1
UCC118	2.13	33.04%	2101	Human	GCF_000008925.1
2D	1.98	33.18%	1905	Horse	GCF_013487885.1
BNS11	2.30	32.98%	2225	Swine	GCF_021266585.1
JCM1046	2.32	32.99%	2295	Swine	GCF_000758365.1
ZLp4b	2.31	32.90%	2252	Swine	GCF_014841055.1
ZLS006	2.18	33.21%	2096	Swine	GCF_002162055.1

### Pan-genome analysis of *L. salivarius* strains

**Fig. 3:**
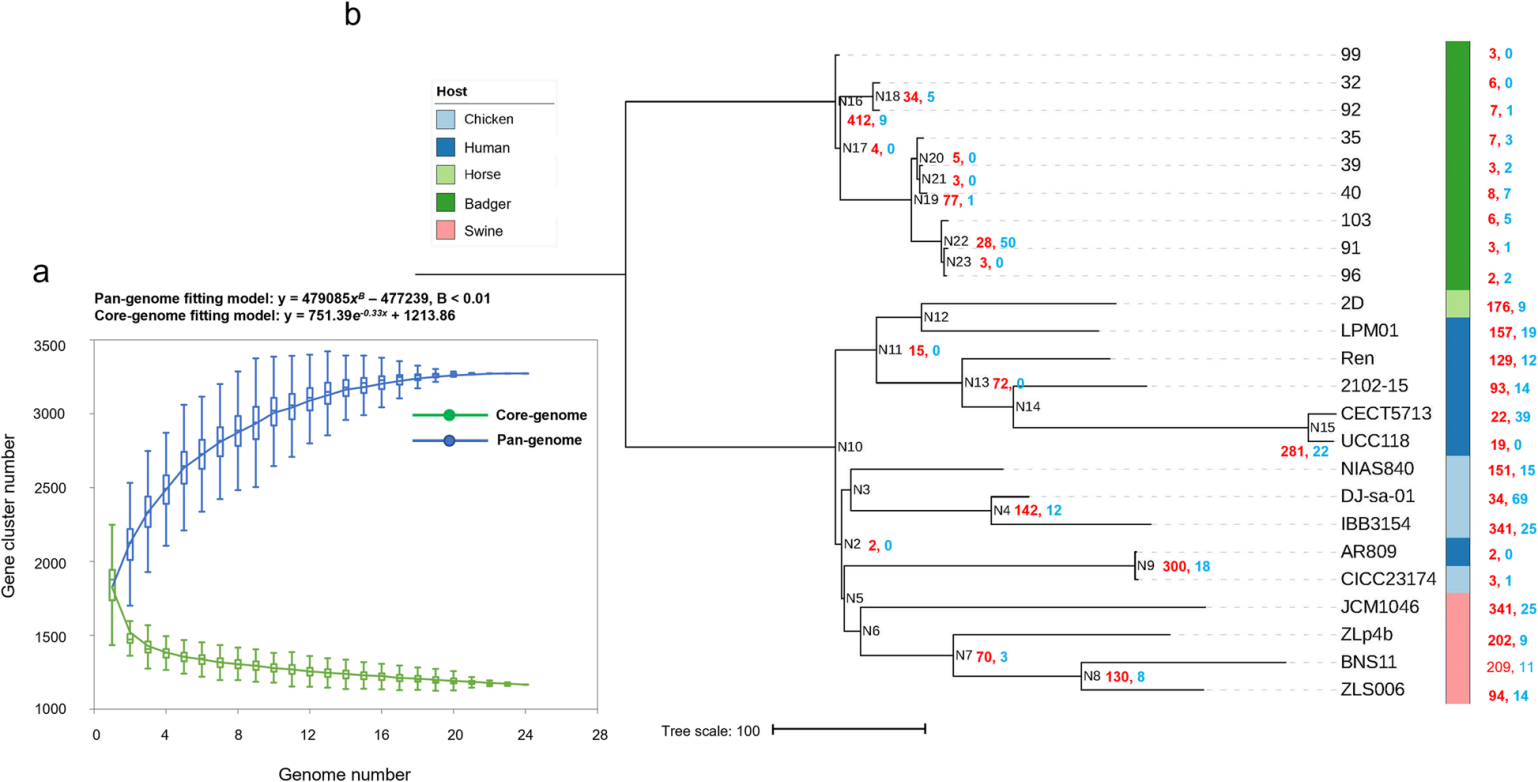
**a** Estimation of the *L. salivarius* pan- and core-genome size. **b** The gene gain and loss events identified by GLOOME analysis. The red colored numbers labeled on the nodes denote the gain events, while the blue colored numbers denote the gene loss events. The tree was constructed based on the gene presence/absence matrix of the pan-genome, and the scale bar denotes 100 gene differences

To identify the genomic regions associated with the host adaptation and/or specificity of *L. salivarius* strains, comparative genomic analyses were performed using the 24 *L. salivarius* genomes. The pan-genome contained 3272 orthologous clusters, whereas the core genome contained 1165 orthologous clusters. The pan-genome of the 24 *L. salivarius* strains was closed, and the pan-genes slowly increased and tended to reach a plateau when the genome number reached 21 (Fig. 3a), suggesting that the 24 genomes could comprehensively represent the total gene repertoire of the *L. salivarius* population from the given hosts. The pan-genome phylogenetic tree constructed based on the gene presence/absence patterns demonstrated that the nine strains originating from badgers differed from each other in terms of the gene contents (Fig. 3b). The three strains, S35, S39, and S40, which were isolated from the same badger individual exhibited relatively conserved genomic contents and formed a cluster in the phylogenetic tree, and S103, S91, and S96 that were isolated from another badger individual also formed a separate cluster; however, the two strains isolated from different individuals, S32 and S92, formed a cluster, and S99 harbored a relatively divergent genomic content compared with the other eight strains. Of note, the badger-originated strains formed a distinct clade from the other strains, clearly suggesting the presence of badger-specific gene families (Fig. 3b). To identify genes that may contribute to the observed host range differences among the strains, gene gain/loss analysis was performed using the GLOOME server. In total, 3413 gene gain events and 392 gene loss events were identified across the phylogenetic tree (Fig. 3b). Notably, while only a minute fraction of gene gain events occurred in the strains originating from badgers, their common ancestor (internal node N16) gained 412 genes but lost only nine genes, suggesting the importance of these gained genes in its adaptation to this wild host.

A total of 270 out of the 412 genes obtained functional annotations, and these COG-annotated genes were mainly assigned to the COG categories “L” (replication, recombination, and repair; 12.22%), “K” (transcription; 11.85%), “M” (cell wall/membrane/envelope biogenesis; 12.22%), and “G” (carbohydrate transport and metabolism; 11.48%) (Table S1). Of note, more than half of the genes in the category “M” (57.58%) encoded glycosyl transferases (GTs), which are involved in the metabolism and transport of carbohydrate substrates. In total, 51.61% of the genes in the category “G” encoded phosphoenolpyruvate–phosphotransferase systems (PEP–PTSs), which are associated with transporters responsible for the uptake of many types of carbohydrate nutrients, including sucrose, fructose, and glucose. We speculated that these gained functional genes could contribute to the adaptation of *L. salivarius* to the intestinal environment of badgers, in which diverse glycan-rich resources are present because of the complex food consumption behavior of badger individuals in the wild.

Positive selection is known as an important driving force for microbes in the niche adaptation process [34]. We further identified the positive selection-affected genes among the badger clade-gained genes using BUSTED [35], and 14 genes were found to be affected by positive selection (*P* < 0.05). Interestingly, two GT-encoding genes among the gained genes that belonged to the COG category “M” were under positive selection, indicative of the importance of the gained GT-encoding genes for the adaptation of *L. salivarius* to wild badgers. Furthermore, *cas9*, whose product is a crucial component in the IIA system and is responsible for recognizing and cleaving subsequent invading nucleic acids with sequences identical to that of the spacer [36], was found to be gained by the badger clade and affected by positive selection.

### CAZymes

**Fig. 4:**
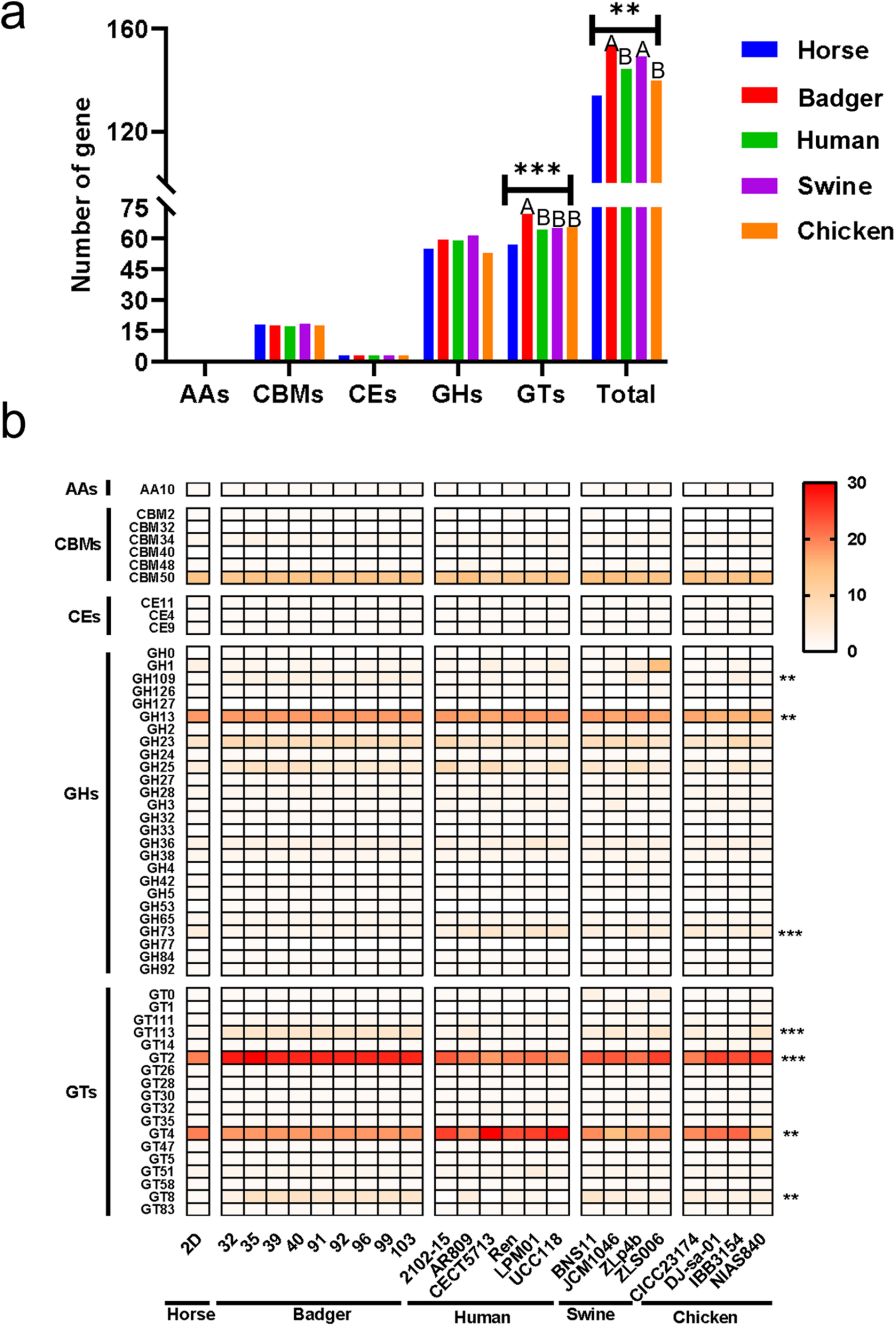
Genes encoding CAZymes in the genome of *L. salivarius* strains among different hosts. **a** Distribution and abundance of CAZymes categories among different hosts. Kruskal–Wallis test with Mann–Whitney post hoc test was used for comparisons. The compact letter display indicates significant differences in pairwise comparisons, and groups with different letters are significantly different (*P* < 0.05). **b** Heatmap of the number of specific CAZymes categories in the genome of *L. salivarius* strains from different hosts. ** denotes *P* < 0.01 and *** denotes *P* < 0.001 based on Kruskal–Wallis test, and the Mann–Whitney post hoc test results were shown in Table S4. The horse group which contains only one strain was excluded from the statistical analysis

Carbohydrate utilization-associated genes were then annotated from the *L. salivarius* genomes using dbCAN2, and one auxiliary activity (AA) family, three carbohydrate esterase (CE) families, six carbohydrate-binding module (CBM) families, 26 glycoside hydrolase (GH) families, and 18 GT families were identified. Genes belonging to GHs and GTs were the most abundant gene families in the 24 *L. salivarius* strains. Notably, the number of annotated CAZyme-encoding genes significantly differed among the strains from different hosts. The strains originating from badgers harbored the highest number of CAZyme-encoding genes, which was significantly higher than that of the human and chicken groups (Fig. 4a) (Kruskal–Wallis test with Mann–Whitney post hoc test, both *P* < 0.01). GHs are responsible for catalyzing the hydrolysis of glycosidic bonds between carbohydrates or carbohydrates and non-carbohydrates [37]. GH13, GH23, and GH25 were the most abundant families in 24 genomes. Interestingly, the amounts of GH13-, GH109-, and GH73-encoding genes significantly varied among the strains from different hosts (Kruskal–Wallis test, all *P* < 0.05). While greater amounts of GH13- and GH109-encoding genes were identified in the strains from badgers than in those from other hosts, a significantly lower amount of GH73-encoding genes was identified in the strains from badgers than in those from other hosts (Fig. 4b) (all *P* < 0.01, Mann–Whitney test).

GTs are enzymes that catalyze the formation of various glycoconjugates [38]. Of these, GT2, GT4, GT8, and GT113 represent the most predominant GT family members in the 24 *L. salivarius* strains (Fig. 4c). Notably, the number of GT-encoding genes significantly differed among the strains from different hosts (Kruskal–Wallis test, *P* = 0.001), with the strains from badgers harboring the highest number of GTs (all *P* < 0.01, Mann–Whitney test). In particular, the amounts of GT2-, GT8-, and GT113-encoding genes significantly differed among the strains from different hosts (Kruskal–Wallis test, all *P* < 0.01), with the strains from badgers harboring the highest amount of all three gene families (all *P* < 0.05, Mann–Whitney test).

### MGEs and the CRISPR–Cas system

**Fig. 5:**
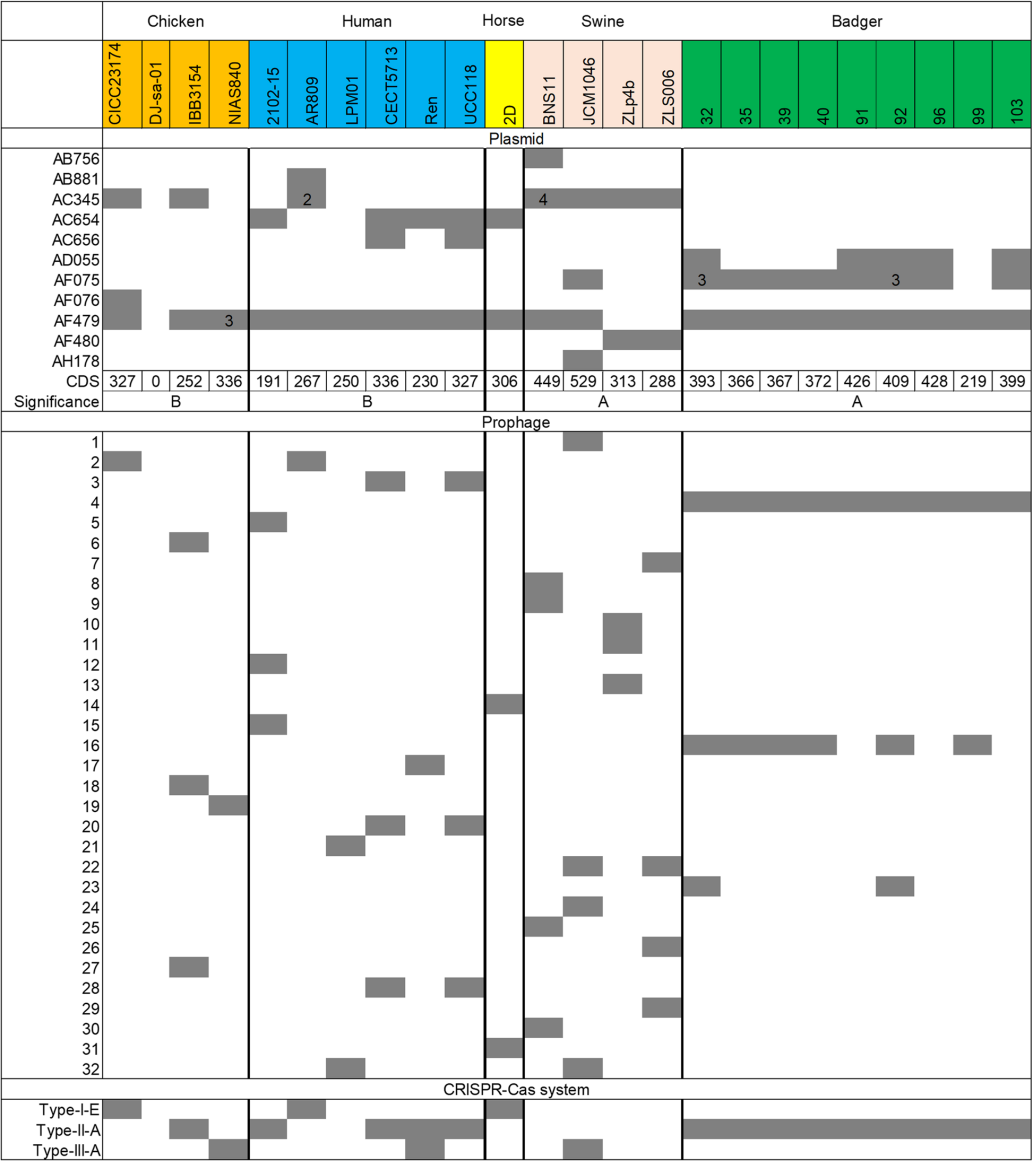
Summary of mobile elements (plasmids and phages) and CRISPR-Cas systems present in *L. salivarius* strains. Upper panel, the colored cells denote the presence of each of the plasmid clusters, and a number is shown in the cell if a strain harbor more than one plasmid in the same group. The plasmid sequences for the strains were grouped into clusters using the mob_type method implemented in mob-suite, and the clusters were named with the primary_cluster_id values deposited in the mob-suite database. The number of CDSs predicted in the plasmid sequences of each strain was also shown, and different letters denote significant differences (*P* < 0.05, Kruskal–Wallis test with Mann–Whitney post hoc test). Middle panel, the colored cells denote presence of each of the prophage clusters. The prophage sequences were grouped into clusters using cd-hit-est with parameter -c 0.9. Lower panel, the colored cells denote presence of each of the three CRISPR-Cas systems

MGEs, such as plasmids and prophages, play a prominent role in shaping a strain’s genome [39] and serve as an important driving force in the evolution of organisms through inter-organism exchanges [40]. Zero (strain DJ-sa-01) to six plasmids were identified from the 24 genomes (Fig. 5). Although the number of plasmids did not vary much among the strains from different hosts (on average, 2–3.5 plasmids per group), the total length of plasmids in the strains from badgers was significantly longer than that in the strains from humans and chickens [346.71 ± 53.53 kb (mean ± SD), 258.40 ± 49.54 kb, and 230. 41 ± 159.97 kb for the strains from badgers, humans, and chickens, respectively] (Mann–Whitney test, *P* < 0.05 for both badgers vs. human and badgers vs. chicken strain comparisons). However, the total length of plasmids in the strains from badgers did not differ significantly from that in the strains from swine (377.21 ± 97.05 kb) (Mann–Whitney test, *P* > 0.05). The number of plasmid-originating CDSs in the strains from badgers was also significantly higher than that in the strains from humans and chickens (Mann–Whitney test, both *P* < 0.05) and did not differ dramatically from that in the strains from swine (*P* > 0.05). The 65 plasmid sequences from the 23 strains (DJ-sa-01 did not harbor plasmid) were grouped into 11 plasmid clusters, with cluster “AF479” observed in the majority of the 23 strains and cluster “AF075” identified in eight out of the nine badge-originated strains but rarely observed in strains originating from other hosts. Of the 412 gene families gained by the badger clade, 110 families originated from plasmids, which included several carbohydrate utilization-associated genes. Among them, an *iol* operon that is involved in the utilization of the important plant metabolite myoinositol (MI), including *iolRABCDG2G3E*, was identified in the plasmids belonging to cluster “AF479” of the nine strains from badgers but was absent in the other strains, except for strain IBB3154, a chicken isolate. The *iol* operon in the nine badger-originated strains was located in a genomic region affected by horizontal gene transfer (HGT) as revealed by the Alien Hunter analysis (Fig. S1).

In total, 52 prophage sequences were identified from the 24 *L. salivarius* genomes, ranging from zero (strain DJ-sa-01) to four prophages identified in the 24 genomes (Fig. 5). The number of prophage sequences significantly varied among the strains from different hosts (Kruskal–Wallis test, *P* = 0.018). The strains from swine (3.75 ± 0.5) were found to harbor significantly more prophage sequences than those from chickens (1.25 ± 1.26), humans (2.17 ± 0.98), and badgers (1.89 ± 0.78) (Mann–Whitney test, all *P* < 0.05), while the number of prophages in the three groups did not differ significantly among each others (*P* > 0.05). These prophage sequences were then clustered into 32 prophage groups (Fig. 5). Notably, prophage cluster 4 was present in all nine strains originating from badgers and cluster 16 was present in seven of the nine strains; however, the two prophage clusters were absent in the remaining 15 strains (Fig. 5), indicating that these two prophages were specifically acquired by the last common ancestor of the badger population after it was divergent from that of the remaining 15 strains. The remaining prophage clusters were found to be strain-specific and highly diverse, suggesting that the prophage acquisition events occurred independently after these strains diverged from their last common ancestor. In total, 83 genes were predicted from the prophage sequences among the 412 genes gained by the common ancestor of badgers; these included certain genes that were beneficial to the bacterial host. For example, a UDP-N-acetyl-D-mannosamine dehydrogenase-encoding gene, which is involved in the biosynthesis of UDP-acetamido sugars, was identified. Furthermore, a cysteine protease-encoding gene, which plays a major role in the degradation of proteins into peptides, amino acids, ammonia, and other forms of nonprotein nitrogen [41], was identified.

The CRISPR–Cas system confers adaptive immunity to host bacteria to resist the insertion of foreign genes, such as phages and plasmids [42]. The CRISPR–Cas system was identified in 79.17% (19/24) of the strains (Fig. 5). Three CRISPR–Cas system types (types I, II, and III) were detected, and one subtype (IE, IIA, and IIIA) was identified for each type. Only one out of the four swine-originated strains was found to harbor the CRISPR–Cas system, which was consistent with the observation that the strains originating from swine harbored a significantly higher number of prophages, as mentioned above. Notably, the type IIA system was identified in 14 out of the 24 strains, and all nine badger-originated strains and four out of six human-originated strains harbored the type IIA system. Furthermore, two genes involved in the CRISPR-Cas system, including *cas2* and *cas9*, were found to be gained by the badger group and affected by positive selection. Spacers are small fragments of foreign DNA incorporated into CRISPR loci to confer adaptive immunity to the host [43]. In total, 412 spacer sequences were obtained from all CRISPR loci of the 24 strains, and 0–89 spacers were identified in each isolate. The number of spacers harbored by the strains did not exhibit obvious host-specific patterns but instead exhibited highly isolate-specific patterns. The spacers that shared identical sequences were defined as a spacer operational taxonomic unit (OTU); 290 OTUs were obtained (Table S2). Of the 290 OTUs, 78.97% (229/290) contained only one spacer sequence. Of the remaining 61 OTUs, only 25 OTUs contained spacers derived from more than two different strains from the same host, badgers. Furthermore, these OTUs grouped the nine badger-originated strains into two clusters (Fig. S2 and Table S2). In cluster 1, four strains from different badger individuals (S91, S92, and S99 from the same badger individual and S32 from another badger individual) shared identical spacer distribution patterns, and S35 exhibited different spacer contents from the other four strains. In cluster 2, S39 and S40, which were isolated from the same badger individual harbored identical spacer contents, and S96 and S103 that were isolated from another individual harbored the same spacer contents (Fig. S2). Subsequently, spacers from all CRISPR loci were extracted and further mapped to the 52 predicted prophage sequences, and only 26 of the 290 OTUs were paired with the prophage sequences (Table S3). The majority of spacers from badgers, as well as spacers from other hosts, did not match their prophage contents, indicating the presence of highly diverse foreign *L. salivarius* phages in the intestinal environments.

## Discussion

LAB are gut commensals that contribute to the maintenance of gut homeostasis by maintaining a beneficial microbial balance [9]. Of the LAB species, *L. salivarius* is an important taxon in the intestinal tracts of mammals. Many LAB species have exhibited promising probiotic properties, such as preventing and controlling infectious diseases [5, 18], modulating the immune system [17, 44], and enhancing the quality of livestock products [45]. Compared with domesticated animals, the gut microbiota of wild animals probably contains certain probiotic members that can balance the negative effects of the consumed pathogen-infected, poisonous foods and resist the invasion of pathogens, thereby maintaining host health [3]. Therefore, wild animals can serve as a promising reservoir to identify probiotic bacteria, such as those affiliated with *L. salivarius*. In this study, all nine *L. salivarius* strains originating from badgers exhibited good intestinal adaptation ability, as revealed by acid and bile tolerance assays and auto-aggregation assays. Furthermore, several strains were effective in controlling the negative effects of intestinal bacterial pathogens on the hosts by reducing the population of pathogens in the intestinal environment through their antagonistic activities (anti-*Salmonella* assay) and coaggregation activities and by preventing the adhesion of pathogens to epithelial cells, which is the first step in pathogen infection [46] (Fig. 1). The aforementioned beneficial effects varied among the nine strains. Overall, strain S32 exhibited good probiotic properties and may thus have an application prospect for controlling infective pathogens, such as *Salmonella*, in animal breeding and raising practices.

Given that badger individuals exhibit a complex feeding habit and consume many types of plant-derived foods as well as other animal-derived foods in the wild [7], which probably contain more complex carbohydrate and other nutrients compared with that are consumed by domesticated animals and humans, the genomes of *L. salivarius* strains originating from badgers were compared with those originating from domesticated animals and humans, aiming to reveal potential beneficial genes in badger-originated *L. salivarius* strains, such as those associated with complex carbohydrate digestion. The whole genome sequences of the nine *L. salivarius* strains originating from badgers were obtained based on a combination of long and short reads and were a significant addition to the number of genomes available in the public database. Certain bacterial species that are widely distributed in various environments usually have an open pan-genome, in which new genes can confer beneficial effects for environmental adaptation [47]. On the other hand, species that tend to occupy limited habitats frequently have a closed pan-genome because they do not need to respond to many environmental changes [48, 49]. Our results demonstrated that *L. salivarius* species harbor a closed pan-genome, similar to that in *L. ruminis* [50]. Previous analyses of *Lactobacillus* species, such as *L. reuteri* [51] and *L. ruminis* [50], consistently suggested that strains from different hosts usually form separate phylogenetic clusters, which are highly reflective of the host source. Notably, the strains originating from badgers harbored a relatively larger genome size and more CDSs and formed a distinct clade from the strains originating from other hosts. This finding indicates that the presence of clade-specific genes contributes to the adaptation of *L. salivarius* to wild badgers, which has a complex feeding habit.

The degradation of complex carbohydrate substrates, such as starch, cellulose, and MI, to simple sugars, is crucial for the metabolism of the bacteria inhabiting the intestine and the hosts and is mainly catalyzed by carbohydrate utilization-associated enzymes [52]. In this study, *L. salivarius* strains from the same host showed highly consistent carbohydrate metabolism behavior, as revealed by CAZyme analysis. Five major CAZymes (AAs, GHs, GTs, CEs, and CBMs) were annotated in the 24 strains. GHs and GTs were the most abundant gene families in *L. salivarius*, with GT2, GT4, GH13, and CBM50 being the largest families, which is consistent with the results in other *Lactobacillus* species [43]. The diet of the host is a key evolutionary force that shapes the gut microbiota and influences the evolutionary trend of gut symbionts [53]. Notably, significantly lower numbers of GH73-encoding genes and higher numbers of GH13- and GH109-encoding genes were noted in the strains from badgers than in those from other hosts (Fig. 4). GH13 and GH109 are known to be crucial for the degradation of several carbohydrates, such as starch, sucrose, and O-glycan [54]. In contrast, GH73 is an important enzyme family involved in the degradation of beta-glucan [54], which is an abundant ingredient in cereals [55]. These findings collectively reflect the difference in food sources between wild badgers and domesticated animals and humans. Gene gain events were suggested to play an important role in the acquisition of complex carbohydrate degradation-associated genes in the evolutionary history of strains from badgers (Table S1). For instance, the *iol* operon, which is involved in the utilization of the important plant metabolite MI, is not widely distributed in the *Lactobacillus* genus [56]; however, all nine strains from badgers harbored the plasmid-derived cargo genes *iolRABCDG2G3E*, which were probably acquired by these strains via HGT and can benefit the bacteria and the host by digesting the plant-derived MI [56]. GT2 and GT8 families are involved in the biosynthesis of exopolysaccharides and capsular polysaccharides, respectively [57]. Polysaccharide production is known to be an important beneficial trait of probiotic *Lactobacillus* species [58]. The higher abundance of GT2 and GT8 families in the strains from badgers may benefit the host by regulating gut microbiota homeostasis through the production of polysaccharides [59]. Besides plasmids, prophages are an important source of the gene gain events which play a critical role in bacterial population evolution [49]. Most of the prophage sequences identified in these badger-originated strains did not show high similarity to other analyzed *L. salivarius* genomes (Fig. 5) and genomes deposited in the NCBI database, these sequences are probably acquired from the microbial members in the badger gut microbiome, which has not been explored yet. Several cargo genes that were probably beneficial for *L. salivarius* to adapt to the badger host, such as the UDP-acetamido sugar biosynthesis-associated gene and cysteine protease-encoding gene, were identified in the prophages specifically harbored by the badger-originated strains. UDP-acetamido sugar biosynthesis-associated gene is considered to be important for bacteria to adapt to extreme environments [60]. The cysteine protease-encoding gene, whose product plays a major role in the degradation of proteins into peptides, amino acids, ammonia, and other forms of nonprotein nitrogen [41], could benefit the badger host by contributing to the utilization of protein contents in the consumed complex foods. Overall, the genomes of *L. salivarius* strains originating from badgers serve as valuable resources to identify probiotic-associated genes, such as those involved in complex carbohydrate substrate degradation and exopolysaccharide production. The CRISPR–Cas system serves as an immune system to prevent the bacterial host from infections caused by foreign DNA, such as phages [42]. In our study, 79.17% (19/24) of the *L. salivarius* strains contained at least one CRISPR–Cas system (Fig. 5). Notably, the *L. salivarius* strains that lacked the CRISPR–Cas system contained more prophages than those possessing the CRISPR–Cas system; a similar result has been reported in *L. ruminis* [50], highlighting the important role of the CRISPR–Cas system in preventing the invasion of exogenous DNA in *Lactobacillus* species. The type II CRISPR-Cas system could play a prominent role in preventing the invasion of foreign DNA during the evolution of *L. salivarius*, particularly for the badger group (all nine strains containing the IIA system). The presence of highly diverse spacers in the *L. salivarius* genomes and the fact that the *cas9* gene (which encodes the crucial component of the type II system and is responsible for recognizing and cleaving subsequent invading nucleic acids with sequences identical to that of the spacer [36]) was under positive selection, collectively indicate the presence of fierce competitions and coevolution between the phages and *L. salivarius* strains.

## Conclusions

Overall, we obtained nine *L. salivarius* strains from the fecal samples of wild badgers individuals with certain probiotic traits and identified an isolate exhibiting overall good performance. Genome sequencing and comparative genomic analysis demonstrated that the strains originating from badgers harbored a larger genome size and a higher number of CDSs than those originating from chickens, humans, and horses and had significantly more genes responsible for the degradation of complex carbohydrate substrates. To some extent, these differences reflect the niche adaptation of *L. salivarius* in badgers through gene acquisition. However, our study also has certain limitations raised by the small number of strains from each host species (1 to 9 strains) due to the limited amount of complete *L. salivarius* genomes available in the NCBI database, and further large-scale comparative genomics analysis is needed in future studies to reveal more comprehensive results. Nevertheless, our findings are an important addition to the *L. salivarius* database and provide new insights into the adaption of *L. salivarius* to distinct hosts from the perspective of evolution, as well as contribute to the probiotic development practices.

## Methods

### Sampling collection

Fresh fecal samples from eight wild badgers individuals were collected from Shanghai, China (30.92 N, 121.46 E), on August 20^th^, 2020. Badgers usually defecate in their fixed locations from midnight to early morning. Only the central inner layer of the feces was collected using aseptic sticks to avoid environmental contamination. The samples were collected into 50 mL sterile tubes and immediately transported to the laboratory on ice.

### Isolation of LAB from fecal samples

In total, 20 g of each fecal sample was homogenized in phosphate-buffered saline (PBS) to prepare serial dilutions. Specifically, the homogenized fecal samples were diluted to 10^−3^, 10^−4^, and 10^−5^, and 200 μL of each dilution was coated onto MRS agar plates (Guangdong Huankai Microbial Technology Co., Ltd, Guangdong, China) for LAB isolation. After 24 h of incubation at 37°C, different types of colonies were selected for further purification based on the colony size, morphology, color, and surface roughness. Purified single colonies were subjected to Gram staining and microscope-based cell morphology determination. The checked purified strains were stored at − 80°C using 25% glycerin as the cryoprotective agent.

### Taxonomic identification of LAB by 16S rRNA gene sequencing

DNA was extracted from each isolate using a DNA extraction kit (Tiangen Biotech Co. Ltd., Beijing, China), according to the manufacturer’s protocol. The DNA concentration and quality were assessed using NanoDrop (Thermo Scientific, USA). The 16S rRNA gene was amplified using the universal primer set 27F (5′-AGAGTTTGATCCTGGCTCAG-3′) and 1492R (5′-GGYTACCTTGTTACGACTT-3′) [61]. The PCR products were further verified by 1% agarose gel electrophoresis, and those with correct size (approximately 1,500 bp) were subjected to 16S rDNA sequencing (Genscript, Nanjing, China). The taxonomic affiliation of these LAB strains was determined based on 16S rRNA gene data of type strains deposited in EzBioCloud database [62]. Of the 91 strains obtained from badger feces, nine strains identified as *L. salivarius* were selected for further experiments and genome sequencing. The nine strains were isolated from two individuals, while strains S32, S35, S39, and S40 were isolated from the same individual, S91, S92, S96, S99, and S103 were from another individual.

### Antagonistic activities of* L. salivarius* strains against *Salmonella* species

The anti-*Salmonella* effects of *L. salivarius* strains were determined using the agar spot test, as described by Schillinger [63], with a pathogenic strain *S.* Derby 14T originating from swine serving as an indicator [64]. After incubation in MRS liquid medium at 37°C for 24 h, 10 µL of each *L. salivarius* culture was inoculated in the center of MRS agar plates, air dried, and incubated at 37°C for an additional 24 h. Following this, 10 µL of overnight grown *S.* Derby 14T culture was added to Luria–Bertani (LB) semi-solid medium and mixed well. The semi-solid medium was then spread on MRS agar plates containing LAB until coagulation. The plates were incubated at 37°C for 24 h, and the diameters of inhibition zones were then measured by subtracting the diameter of the *L. salivarius* colony from the diameter of the measured inhibition zone.

The data from different strains were standardized using the Z-score method. The Z-score was calculated using the formula z = (x − µ)/σ, where x is the diameter of the inhibition zone of each *L. salivarius* isolate; μ denotes the mean value of the overall inhibition zone diameter; and σ is the standard deviation of the overall inhibition zones.

### The anti-adhesion ability of *L. salivarius* strains against the adhesion of *S.* Derby 14T

The mouse colon cancer epithelial cell line MC38 was used in this assay, and it was purchased from Hunan Fenghui Biotechnology Co., Ltd. (Hunan, China) (catalog no. CL0203). The cells were seeded into 24-well plates to a concentration of 4 × 10^5^ cells per well and were cultured overnight at 37°C in the presence of 5% CO_2_. Then, 1 × 10^7^* L. salivarius* cells were added into each well and incubated for 1 h. The cells were then washed with PBS and transferred to a new medium. *S.* Derby 14T cells were added to *L. salivarius-*treated and untreated MC38 cells at a multiplicity of infection (MOI) of 20:1 and incubated for 1 h. The cultured cells were washed using PBS thrice and then solubilized with 0.1% Triton X-100 for 5 min. The solution was then serially diluted, and 100 μL of the 10^−3^ and 10^−4^ diluted samples were evenly coated onto preconfigured sterile LB plates. The plates were incubated at 37°C for 12–16 h to calculate the number of adhered *S.* Derby 14T strains. Three replicates were performed for each treatment. The adhesion rate reduction ratio (%) of *S.* Derby 14T cells to MC38 cells in the presence of *L. salivarius* strains was calculated as follows: [(the number of *S.* Derby 14T cells adhered in the absence of *L. salivarius* strains − the number of *S.* Derby 14T cells adhered in the presence of *L. salivarius* strains)/the number of *S.* Derby 14T cells adhered in the absence of *L. salivarius* strains] × 100%.

### Acid and bile tolerance assays of* L. salivarius* strains

The nine *L. salivarius* strains were grown in 5 mL MRS broth and anaerobically incubated at 37°C for 24 h. The cells were then centrifuged at 4,000 rpm for 10 min, and the obtained bacterial pellet was washed thrice and resuspended to a concentration of OD_600_ 0.8 in sterile PBS. Then, the pH of the bacterial suspensions was adjusted to 3 using 5M HCl, and these suspensions were incubated at 37°C for 0, 2, and 4 h, respectively. Following this, 100 μL aliquots of the bacterial suspensions were serially diluted, and 100 μL of the diluted samples were spread on MRS agar plates and anaerobically incubated at 37°C for 24 h. The number of surviving cells was calculated using the plate counting method [65]. Three replicates were performed for each *L. salivarius* isolate.

For the bile tolerance assay, *L. salivarius* strains were cultured in MRS broth containing 0.15% ox gall. The culture without ox gall was used as the control. The cultures were anaerobically incubated at 37°C for 0, 2, and 4 h. The number of surviving cells was calculated using the plate counting method as mentioned above. Three replicates were performed for each *L. salivarius* isolate.

The tolerance rate of *L. salivarius* strains to acidic pH and bile (%) was calculated using the following formula: [the number of viable bacteria at 2 and 4 h (CFU/mL)/the number of viable bacteria at 0 h (CFU/mL)] × 100%.

### Auto-aggregation ability

The auto-aggregation ability of *L. salivarius* strains was assessed as described previously, with slight modification [66, 67]. The *L. salivarius* strains were grown in MRS broth and anaerobically incubated at 37°C for 24 h. The cells were then centrifuged at 4,000 rpm for 10 min, and the bacterial pellets were washed thrice and resuspended to a concentration of OD_600_ 0.8 in sterile PBS. Following this, 1 mL samples of the bacterial suspensions were well mixed by vertexing, and then were incubated at 37°C without shaking for 5 h. 100 μL of the upper suspension was carefully recovered from the bacterial suspensions and transferred to microtiter plates. Its absorbance was measured using a spectrophotometer (Eppendorf SE Hamburg, Germany) at 600 nm. Three replicates were performed for each *L. salivarius* isolate. The auto-aggregation rate (%) was calculated using the following formula: 1 − (A_t_/A_0_) × 100%, where A_t_ is the absorbance at time t = 5 h and A_0_ is the absorbance at time t = 0 h.

### Coaggregation ability with *Salmonella* strains

The coaggregation ability of *L. salivarius* strains with *Salmonella* strains was assessed as reported previously, with slight modification [68]. Overnight cultures of *L. salivarius* and *S.* Derby 14T were collected by centrifugation at 13,000 rpm for 5 min, and the precipitate was washed thrice and resuspended in sterile PBS. *L. salivarius* and *S.* Derby 14T suspensions were mixed in equal volumes, vortexed for 5 min, and incubated at room temperature [69]. The upper part of the mixture was carefully removed, and its OD_600_ value was measured at 5 h. Single suspensions of each strain were considered as controls. Three replicates were performed for each *L. salivarius* isolate. The coaggregation rate (%) was calculated using the following formula: [(A_x_ + A_y_)/2 − A_(x + y)_]/(A_x_ + A_y_)/2 × 100%, where A_x_, A_y,_ and A_(x + y)_ represent the OD_600_ values of *S.* Derby 14T strains, *L. salivarius* strains, and the *S.* Derby 14T and *L. salivarius* mixture, respectively.

### Genomic DNA extraction, whole genome sequencing, and genome assembly

The genomic DNA of nine *L. salivarius* strains was extracted using the QIAamp® PowerFecal® DNA Kit (Qiagen, Germany), according to the manufacturer’s protocol. The DNA concentration and integrity were assessed using NanoDrop and 0.8% agarose gel electrophoresis, respectively. Then, the DNA concentration was measured using Qubit 4.0 (Thermo Fisher Scientific, USA) to ensure that it satisfied the library preparation.

Whole-genome sequencing was performed on an Illumina HiSeq platform and a Nanopore platform to generate both short and long reads. High-quality long reads (Q > 7 and length > 1 kb) were assembled using Flye (v2.8.3) [70]. The generated assemblies were then polished using NextPolish (v2) [71], with the short reads being used as inputs. The genomic sequences of the nine *L. salivarius* isolates have been deposited in the NCBI database under the accession numbers CP114503- CP114529 (Table 1).

### Comparative genomics analysis

Fifteen completely sequenced *L. salivarius* genome sequences available in the NCBI database (as of March 10^th^, 2022) were downloaded, and the nine de novo assembled genomes and 15 downloaded genomes were simultaneously annotated using Prokka (v1.14.6) with default parameters [72]. Following this, the Clusters of Orthologous Groups of proteins (COG) annotations were generated using eggNOG-mapper (v2.1.7) with parameters –itype proteins and -m diamond based on the eggNOG orthology database (v5.0.2) [73]. The carbohydrate-active enzyme (CAZyme)-encoding genes in the *L. salivarius* genomes were annotated using dbCAN (v2) with parameter -t all, and the Prokka-generated protein sequences were used as inputs [74]. The CRISPR–Cas systems and spacer sequences were identified using CRISPRone with default parameters (https://omics.informatics.indiana.edu/CRISPRone/check.php?id=K4KfCLvy) [75]. Moreover, prophages in the *L. salivarius* genomes were identified using ProphET with default parameters, and the genome sequences and the Prokka-generated gff files were used as inputs in the analysis [76]. The information of the identified prophages in the genomes were listed in Table S5. The prophage sequences were clustered to groups using cd-hit-est with parameter -c 0.9 [49, 77]. The plasmids were clustered to groups using the mob_typer method implemented in mob-suite with default parameters [78]. The acquired genomic contents in the genome sequences via horizontal transfer were predicted using Alien Hunter with default parameters [79].

The average nucleotide identity (ANI) values between the *L. salivarius* strains were calculated according to the methods recommended in FastANI (v1.32) with default parameters [80]. The pan-genome and core genome of the 24 strains were constructed using the OMCL algorithm implemented in the get_homologues package (v 3.3.2) with parameters -M -S 75 -t 0 -e [81]. The pan-genome size, as well as the fitted models of the pan-genome and core genome, were determined using the distance guide algorithm implemented in PanGP (v1.0.1) based on the generated orthologous groups by the get_homologues analysis [82]. The fitted models of the pan-genome and core genome profiles were depicted as y1 = A*x*^*B*^ + C and y2 = D*e*^*Ex*^ + F, respectively, where x is the genome number, y1 is the pan-genome size, y2 is the core genome size, and A, B, C, D, E, and F are fitted parameters. The phylogenetic signal-informative core genes were identified using the get_phylomarkers package [83] and were concatenated using Gblocksb0.91 [84]. The phylogenetic tree was then constructed using IQ-tree [85]. The gene gain/loss analysis was performed using GLOOME with default parameters, and the presence/absence matrix of the gene families and the phylogenetic tree served as inputs, and the probability of gain/loss events (PP ≥ 0.8) was estimated using the stochastic mapping method [86]. The results were visualized using iTol [87]. BUSTED [35] in the HYPHY2.5.31 package [88] was used to identify the orthologous groups affected by positive selection (*P* < 0.05), and the analysis was performed with default parameters.

### Supplementary Information

**Additional file 1. **

**Additional file 2. **

## Data Availability

The Illumina and Nanopore sequencing reads of the nine strains have been deposited in the Sequence Read Archive (SRA) database under BioProject accession number PRJNA911709 (https://www.ncbi.nlm.nih.gov/bioproject/?term=PRJNA911709), and the accession numbers for the genome sequences were CP114503- CP114529.

## References

[1] Huang H, Yie S, Liu Y, Wang C, Cai Z, Zhang W, Lan J, Huang X, Luo L, Cai K (2016). Dietary resources shape the adaptive changes of cyanide detoxification function in giant panda (*Ailuropoda melanoleuca*). Sci Rep.

[2] Lanszki J, Hayward MW, Nagyapáti N (2018). Feeding responses of the golden jackal after reduction of anthropogenic food subsidies. PLoS One.

[3] Wang Y, Xu J, Chen H, Yu J, Xu X, Sun L, Xu X, Yu C, Xu F, Huang J (2022). A balanced gut microbiota is essential to maintain health in captive sika deer. Appl Microbiol Biotechnol.

[4] Claesson MJ, Li Y, Leahy S, Canchaya C, van Pijkeren JP, Cerdeño-Tárraga AM, Parkhill J, Flynn S, O'Sullivan GC, Collins JK, Higgins D, Shanahan F, Fitzgerald GF, van Sinderen D, O’Toole PW (2006). Multireplicon genome architecture of *Lactobacillus salivarius*. Proc Natl Acad Sci U S A.

[5] Messaoudi S, Manai M, Kergourlay G, Prévost H, Connil N, Chobert JM, Dousset X (2013). *Lactobacillus salivarius*: bacteriocin and probiotic activity. Food Microbiol.

[6] Hardstaff JL, Marion G, Hutchings MR, White PC (2014). Evaluating the tuberculosis hazard posed to cattle from wildlife across Europe. Res Vet Sci.

[7] Li F, Luo Z, Li C, Li C, Jiang Z (2013). Biogeographical patterns of the diet of Palearctic badger: Is badger an earthworm specialist predator?. Chinese Sci Bull.

[8] Stedman A, Maluquer de Motes C, Lesellier S, Dalley D, Chambers M, Gutierrez-Merino J (2018). Lactic acid Bacteria isolated from European badgers (*Meles meles*) reduce the viability and survival of Bacillus Calmette-Guerin (BCG) vaccine and influence the immune response to BCG in a human macrophage model. BMC Microbiol.

[9] Stedman A, van Vliet AHM, Chambers M, Gutierrez-Merino J (2020). Gut commensal bacteria show beneficial properties as wildlife probiotics. Ann N Y Acad Sci.

[10] Guo XH, Kim JM, Nam HM, Park SY, Kim JM (2010). Screening lactic acid bacteria from swine origins for multistrain probiotics based on in vitro functional properties. Anaerobe.

[11] Kang CH, Han SH, Kim Y, Paek NS, So JS (2018). In vitro probiotic properties of *Lactobacillus salivarius* MG242 isolated from human vagina. Probiotics Antimicrob Proteins.

[12] Messaoudi S, Madi A, Prévost H, Feuilloley M, Manai M, Dousset X, Connil N (2012). In vitro evaluation of the probiotic potential of *Lactobacillus salivarius* SMXD51. Anaerobe.

[13] Raftis EJ, Forde BM, Claesson MJ, O'Toole PW (2014). Unusual genome complexity in *Lactobacillus salivarius* JCM1046. BMC Genomics.

[14] Martín R, Jiménez E, Olivares M, Marín ML, Fernández L, Xaus J, Rodríguez JM (2006). *Lactobacillus salivarius* CECT 5713, a potential probiotic strain isolated from infant feces and breast milk of a mother–child pair. Int J Food Microbiol.

[15] Kang MS, Lim HS, Oh JS, Lim YJ, Wuertz-Kozak K, Harro JM, et al. Antimicrobial activity of *Lactobacillus salivarius* and *Lactobacillus fermentum* against *Staphylococcus aureus*. Pathog Dis. 2017;75(2). 10.1093/femspd/ftx009. 10.1093/femspd/ftx009 28158586

[16] Ryan KA, Daly P, Li Y, Hooton C, O'Toole PW (2008). Strain-specific inhibition of *Helicobacter pylori* by *Lactobacillus salivarius* and other *lactobacilli*. J Antimicrob Chemother.

[17] Zhai Q, Shen X, Cen S, Zhang C, Tian F, Zhao J, Zhang H, Xue Y, Chen W (2020). Screening of *Lactobacillus salivarius* strains from the feces of Chinese populations and the evaluation of their effects against intestinal inflammation in mice. Food Funct.

[18] O'Hara AM, O'Regan P, Fanning A, O'Mahony C, Macsharry J, Lyons A, Bienenstock J, O'Mahony L, Shanahan F (2006). Functional modulation of human intestinal epithelial cell responses by *Bifidobacterium infantis* and *Lactobacillus salivarius*. Immunology.

[19] Gilman J, Cashman KD (2006). The effect of probiotic bacteria on transepithelial calcium transport and calcium uptake in human intestinal-like Caco-2 cells. Curr Issues Intest Microbiol.

[20] Zhao Y, Zhao L, Zheng X, Fu T, Guo H, Ren F (2013). *Lactobacillus salivarius* strain FDB89 induced longevity in *Caenorhabditis elegans* by dietary restriction. J Microbiol.

[21] Fang F, Li Y, Bumann M, Raftis EJ, Casey PG, Cooney JC, Walsh MA, O'Toole PW (2009). Allelic variation of bile salt hydrolase genes in *Lactobacillus salivarius* does not determine bile resistance levels. J Bacteriol.

[22] Harris HMB, Bourin MJB, Claesson MJ, O'Toole PW (2017). Phylogenomics and comparative genomics of *Lactobacillus salivarius*, a mammalian gut commensal. Microb Genom.

[23] Francis F, Kim J, Ramaraj T, Farmer A, Rush MC, Ham JH (2013). Comparative genomic analysis of two *Burkholderia glumae* strains from different geographic origins reveals a high degree of plasticity in genome structure associated with genomic islands. Mol Genet Genomics.

[24] Lee JY, Han GG, Kim EB, Choi YJ (2017). Comparative genomics of *Lactobacillus salivarius* strains focusing on their host adaptation. Microbiol Res.

[25] Cao MD, Nguyen SH, Ganesamoorthy D, Elliott AG, Cooper MA, Coin LJ (2017). Scaffolding and completing genome assemblies in real-time with nanopore sequencing. Nat Commun.

[26] Ashton PM, Nair S, Dallman T, Rubino S, Rabsch W, Mwaigwisya S, Wain J, O'Grady J (2015). MinION nanopore sequencing identifies the position and structure of a bacterial antibiotic resistance island. Nat Biotechnol.

[27] Zhang W, Sun Z, Menghe B, Zhang H (2015). Short communication: Single molecule, real-time sequencing technology revealed species- and strain-specific methylation patterns of 2 *Lactobacillus* strains. J Dairy Sci.

[28] Schmid M, Muri J, Melidis D, Varadarajan AR, Somerville V, Wicki A, Moser A, Bourqui M, Wenzel C, Eugster-Meier E, Frey JE, Irmler S, Ahrens CH (2018). Comparative genomics of completely sequenced *Lactobacillus helveticus* genomes provides insights into strain-specific genes and resolves metagenomics data down to the strain level. Front Microbiol.

[29] Tedersoo L, Albertsen M, Anslan S, Callahan B (2021). Perspectives and benefits of high-throughput long-read sequencing in microbial ecology. Appl Environ Microbiol.

[30] Mallappa RH, Singh DK, Rokana N, Pradhan D, Batish VK, Grover S (2019). Screening and selection of probiotic *Lactobacillus* strains of Indian gut origin based on assessment of desired probiotic attributes combined with principal component and heatmap analysis. LWT.

[31] Tyagi AK, Prasad S (2019). Corrigendum: Commentary: Probiotic and technological properties of Lactobacillus spp. strains from the human stomach in the search for potential candidates against gastric microbial dysbiosis. Front Microbiol.

[32] Kos B, Šušković J, Vuković S, Šimpraga M, Frece J, Matošić S (2003). Adhesion and aggregation ability of probiotic strain *Lactobacillus acidophilus* M92. J Appl Microbiol.

[33] Fattinger SA, Sellin ME, Hardt WD (2021). *Salmonella* effector driven invasion of the gut epithelium: breaking in and setting the house on fire. Curr Opin Microbiol.

[34] Zhang Y, Jalan N, Zhou X, Goss E, Jones JB, Setubal JC, Deng X, Wang N (2015). Positive selection is the main driving force for evolution of citrus canker-causing *Xanthomonas*. Isme J.

[35] Murrell B, Weaver S, Smith MD, Wertheim JO, Murrell S, Aylward A, Eren K, Pollner T, Martin DP, Smith DM (2015). Gene-wide identification of episodic selection. Mol Biol Evol.

[36] Garneau JE, Dupuis MÈ, Villion M, Romero DA, Barrangou R, Boyaval P, Fremaux C, Horvath P, Magadán AH, Moineau S (2010). The CRISPR/Cas bacterial immune system cleaves bacteriophage and plasmid DNA. Nature.

[37] Letsididi R, Letsididi KS, Zhang T, Jiang B, Mu W (2018). Molecular modelling of a thermostable glycoside hydrolase from *Caldivirga maquilingensis* and its substrate docking mechanism for galactooligosaccharides synthesis. Biomedicine.

[38] Na L, Li R, Chen X (2021). Recent progress in synthesis of carbohydrates with sugar nucleotide-dependent glycosyltransferases. Curr Opin Chem Biol.

[39] Syrokou MK, Paramithiotis S, Drosinos EH, Bosnea L, Mataragas M (2022). A comparative genomic and safety assessment of six Lactiplantibacillus plantarum subsp. argentoratensis strains isolated from spontaneously fermented Greek wheat sourdoughs for potential biotechnological application. Int J Mol Sci.

[40] Liu M, Siezen RJ, Nauta A (2009). In silico prediction of horizontal gene transfer events in *Lactobacillus bulgaricus* and *Streptococcus thermophilus* reveals protocooperation in yogurt manufacturing. Appl Environ Microbiol.

[41] Santamaría I, Velasco G, Pendás AM, Paz A, López-Otın C (1999). Molecular cloning and structural and functional characterization of human cathepsin F, a new cysteine proteinase of the papain family with a long propeptide domain. J Biol Chem.

[42] Hidalgo-Cantabrana C, Crawley AB, Sanchez B, Barrangou R (2017). Characterization and exploitation of CRISPR loci in *Bifidobacterium longum*. Front Microbiol.

[43] Zhao Y, Yu L, Tian F, Zhao J, Zhang H, Chen W, Xue Y, Zhai Q (2022). Environment-related genes analysis of *Limosilactobacillus fermentum* isolated from food and human gut: genetic diversity and adaption evolution. Foods.

[44] Zhang J, Deng J, Wang Z, Che C, Li YF, Yang Q (2011). Modulatory effects of *Lactobacillus salivarius* on intestinal mucosal immunity of piglets. Current microbiol.

[45] Chaves B, Brashears M, Nightingale K (2017). Applications and safety considerations of *Lactobacillus salivarius* as a probiotic in animal and human health. J Appl Microbiol.

[46] Yumoto H, Hirota K, Hirao K, Ninomiya M, Murakami K, Fujii H, Miyake Y (2019). The pathogenic factors from oral *streptococci* for systemic diseases. Int J Mol Sci.

[47] Tettelin H, Riley D, Cattuto C, Medini D (2008). Comparative genomics: the bacterial pan-genome. Curr Opin Microbiol.

[48] Lefébure T, Morvan C, Malard F, François C, Konecny-Dupré L, Guéguen L, Weiss-Gayet M, Seguin-Orlando A, Ermini L, Der Sarkissian C (2017). Less effective selection leads to larger genomes. Genome Res.

[49] Yu J, Xu X, Wang Y, Zhai X, Pan Z, Jiao X, Zhang Y (2022). Prophage-mediated genome differentiation of the *Salmonella* Derby ST71 population. Microb Genom.

[50] Wang S, Yang B, Ross RP, Stanton C, Zhao J, Zhang H, Chen W (2020). Comparative genomics analysis of *Lactobacillus ruminis* from different niches. Genes.

[51] Yu J, Zhao J, Song Y, Zhang J, Yu Z, Zhang H, Sun Z (2018). Comparative genomics of the herbivore gut symbiont *Lactobacillus reuteri* reveals genetic diversity and lifestyle adaptation. Front Microbiol.

[52] Smokvina T, Wels M, Polka J, Chervaux C, Brisse S, Boekhorst J, van HylckamaVlieg JET, Siezen RJ. *Lactobacillus paracasei* comparative genomics: towards species pan-genome definition and exploitation of diversity. PloS One. 2013;8(7):e68731. 10.1371/journal.pone.0068731 PMC3716772 23894338

[53] Martino ME, Joncour P, Leenay R, Gervais H, Shah M, Hughes S, Gillet B, Beisel C, Leulier F (2018). Bacterial adaptation to the host's diet is a key evolutionary force shaping *Drosophila*-*Lactobacillus* symbiosis. Cell Host Microbe.

[54] Ausland C, Zheng J, Yi H, Yang B, Li T, Feng X, Zheng B, Yin Y (2021). dbCAN-PUL: a database of experimentally characterized CAZyme gene clusters and their substrates. Nucleic Acids Res.

[55] Ahmad A, Anjum FM, Zahoor T, Nawaz H, Dilshad SMR (2012). Beta glucan: a valuable functional ingredient in foods. Crit Rev Food Sci Nutr.

[56] Yebra MJ, Zúñiga M, Beaufils S, Pérez-Martínez G, Deutscher J, Monedero V (2007). Identification of a gene cluster enabling *Lactobacillus casei* BL23 to utilize myo-inositol. Appl Environ Microbiol.

[57] Altermann E, Russell WM, Azcarate-Peril MA, Barrangou R, Buck BL, McAuliffe O, Souther N, Dobson A, Duong T, Callanan M (2005). Complete genome sequence of the probiotic lactic acid bacterium *Lactobacillus acidophilus* NCFM. Proc Natl Acad Sci U S A.

[58] Patten D, Laws AP (2015). *Lactobacillus*-produced exopolysaccharides and their potential health benefits: a review. Benef Microbes.

[59] Caggianiello G, Kleerebezem M, Spano G (2016). Exopolysaccharides produced by lactic acid bacteria: from health-promoting benefits to stress tolerance mechanisms. Appl Microbiol Biotechnol.

[60] Moon YJ, Kwon J, Yun SH, Lim HL, Kim J, Kim SJ, Kang SG, Lee JH, Kim SI, Chung YH (2015). Proteomic insights into sulfur metabolism in the hydrogen-producing hyperthermophilic archaeon *Thermococcus onnurineus* NA1. Int J Mol Sci.

[61] Delbès C, Ali-Mandjee L, Montel MC (2007). Monitoring bacterial communities in raw milk and cheese by culture-dependent and-independent 16S rRNA gene-based analyses. Appl Environ Microbiol.

[62] Yoon SH, Ha SM, Kwon S, Lim J, Kim Y, Seo H, Chun J (2017). Introducing EzBioCloud: a taxonomically united database of 16S rRNA gene sequences and whole-genome assemblies. Int J Syst Evol Microbiol.

[63] Schillinger U (1989). Antibacterial activity of *Lactobacillus sake* isolated from meat. Appl Environ Microbiol.

[64] Yuan X, Xue H, Xu X, Jiao X, Pan Z, Zhang Y (2022). Closely related *Salmonella* Derby strains triggered distinct gut microbiota alteration. Gut Pathog.

[65] Bao Y, Zhang Y, Zhang Y, Liu Y, Wang S, Dong X, Wang Y, Zhang H (2010). Screening of potential probiotic properties of *Lactobacillus fermentum* isolated from traditional dairy products. Food Control.

[66] Angmo K, Kumari A, Bhalla TC (2016). Probiotic characterization of lactic acid bacteria isolated from fermented foods and beverage of Ladakh. LWT-food Sci Technol.

[67] Collado MC, Meriluoto J, Salminen S (2008). Adhesion and aggregation properties of probiotic and pathogen strains. Eur Food Res Technol.

[68] Handley PS, Harty DW, Wyatt JE, Brown CR, Doran JP, Gibbs AC (1987). A comparison of the adhesion, coaggregation and cell-surface hydrophobicity properties of fibrillar and fimbriate strains of *Streptococcus salivarius*. Microbiology.

[69] Beck BR, Park GS, Lee YH, Im S, Jeong DY, Kang J (2019). Whole genome analysis of *Lactobacillus plantarum* strains isolated from kimchi and determination of probiotic properties to treat mucosal infections by Candida albicans and Gardnerella vaginalis. Front Microbiol.

[70] Kolmogorov M, Yuan J, Lin Y, Pevzner PA (2019). Assembly of long, error-prone reads using repeat graphs. Nat Biotechnol.

[71] Hu J, Fan J, Sun Z, Liu S (2020). NextPolish: a fast and efficient genome polishing tool for long-read assembly. Bioinformatics.

[72] Seemann T (2014). Prokka: rapid prokaryotic genome annotation. Bioinformatics.

[73] Cantalapiedra CP, Hernández-Plaza A, Letunic I, Bork P, Huerta-Cepas J (2021). eggNOG-mapper v2: functional annotation, orthology assignments, and domain prediction at the metagenomic scale. Mol Biol Evol.

[74] Yin Y, Mao X, Yang J, Chen X, Mao F, Xu Y (2012). dbCAN: a web resource for automated carbohydrate-active enzyme annotation. Nucleic Acids Res.

[75] Zhang Q, Ye Y (2017). Not all predicted CRISPR–Cas systems are equal: isolated cas genes and classes of CRISPR like elements. BMC Bioinform.

[76] Reis-Cunha JL, Bartholomeu DC, Manson AL, Earl AM, Cerqueira GC (2019). ProphET, prophage estimation tool: A stand-alone prophage sequence prediction tool with self-updating reference database. PLoS ONE.

[77] Fu L, Niu B, Zhu Z, Wu S, Li W (2012). CD-HIT: accelerated for clustering the next-generation sequencing data. Bioinformatics.

[78] Robertson J, Bessonov K, Schonfeld J, Nash JH (2020). Universal whole-sequence-based plasmid typing and its utility to prediction of host range and epidemiological surveillance. Microb Genom.

[79] Vernikos GS, Parkhill J (2006). Interpolated variable order motifs for identification of horizontally acquired DNA: revisiting the *Salmonella* pathogenicity islands. Bioinformatics.

[80] Jain C, Rodriguez-R LM, Phillippy AM, Konstantinidis KT, Aluru S (2018). High throughput ANI analysis of 90K prokaryotic genomes reveals clear species boundaries. Nat Commun.

[81] Contreras-Moreira B, Vinuesa P (2013). GET_HOMOLOGUES, a versatile software package for scalable and robust microbial pangenome analysis. Appl Environ Microbiol.

[82] Zhao Y, Jia X, Yang J, Ling Y, Zhang Z, Yu J, Wu J, Xiao J (2014). PanGP: a tool for quickly analyzing bacterial pan-genome profile. Bioinformatics.

[83] Vinuesa P, Ochoa-Sánchez LE, Contreras-Moreira B (2018). GET_PHYLOMARKERS, a software package to select optimal orthologous clusters for phylogenomics and inferring pan-genome phylogenies, used for a critical geno-taxonomic revision of the genus *Stenotrophomonas*. Front Microbiol.

[84] Castresana J (2000). Selection of conserved blocks from multiple alignments for their use in phylogenetic analysis. Mol Biol Evol.

[85] Minh BQ, Schmidt HA, Chernomor O, Schrempf D, Woodhams MD, Von Haeseler A, Lanfear R (2020). IQ-TREE 2: new models and efficient methods for phylogenetic inference in the genomic era. Mol Biol Evol.

[86] Cohen O, Ashkenazy H, Belinky F, Huchon D, Pupko T (2010). GLOOME: gain loss mapping engine. Bioinformatics.

[87] Letunic I, Bork P (2019). Interactive Tree Of Life (iTOL) v4: recent updates and new developments. Nucleic Acids Res.

[88] Pond SLK, Muse SV (2005). HyPhy: hypothesis testing using phylogenies. Bioinformatics.

