# Supplementary material for: Assessment of beneficial effects and identification of host adaptation-associated genes of Ligilactobacillus salivarius isolated from badgers
Source: BMC Genomics. 2023 Sep 7;24:530. doi: 10.1186/s12864-023-09623-8 (PMC10483869; doi:10.1186/s12864-023-09623-8)
Supplement: Supplementary file 2 — Additional file 2. [file 12864_2023_9623_MOESM2_ESM.docx]

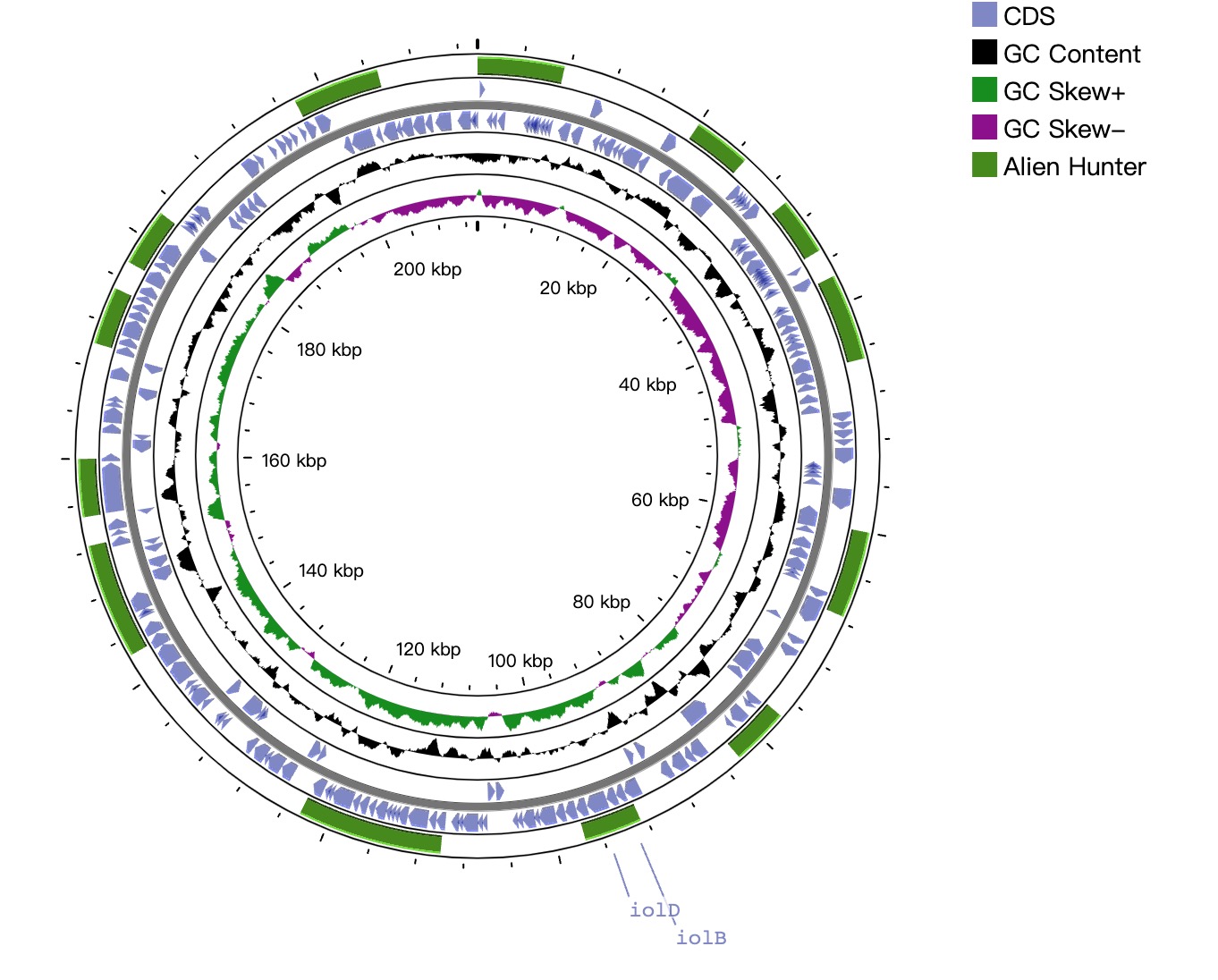


Fig. S1. Circular representation of the *iol* gene cluster-containing plasmid in badger-originated *L. salivarius* strains. The contents of the feature rings (starting with the outermost ring) are as follows: Ring 1, the detected HGT regions by Alien Hunter analysis; Ring 2 and Ring 3, features from the forward and reverse strands, respectively; Ring 4, GC content; Ring 5, GC skew. The plasmid sequences of the nine badger-originated strains were highly conversed and the plasmid from strain S32 was shown.


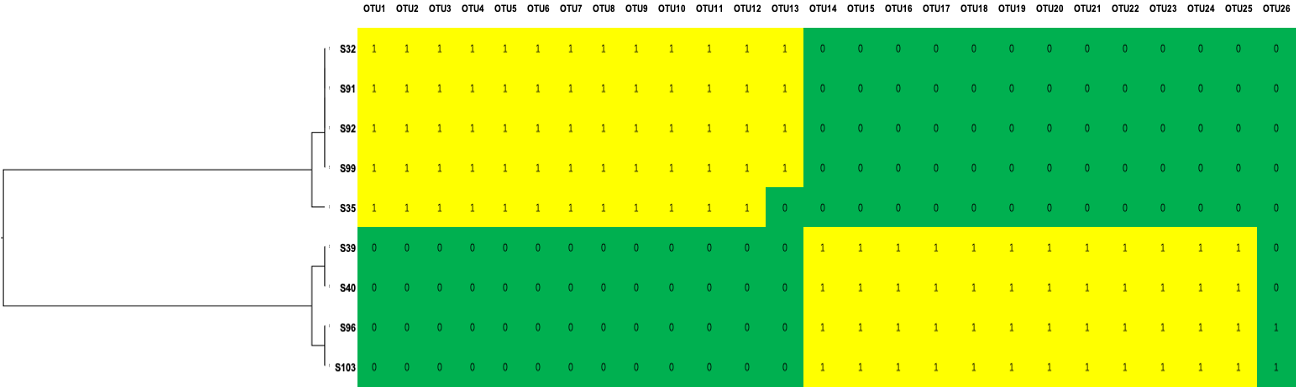


Fig. S2. Hierarchical clustering of nine badger-originated strains based on presence patterns of spacer OTUs. The presence and absence of each OTU are indicated in yellow and green, respectively. The UPGMA tree was generated by using DendroUPGMA (http://genomes.urv.es/UPGMA/) with Dice coefficient.

Supplementary note: the scriptss used in this study.

1. *prokka mygenome.fasta --quiet --outdir outdir/ --prefix mygenome --locustag mygenome*
2. *emapper.py --cpu 36 -i mygenome.faa --itype proteins --usemem --output mygenome -m diamond*
3. *run_dbcan mygenome.faa protein -t all --out_outdir/ output --db_dir /data/dbcan/*
4. *~/ProphET/UTILS.dir/GFFLib/gff_rewrite.pl --input megenome.gff -output mygenome --add_missing_features; ~/ProphET/ProphET_standalone.pl –fasta mygenome.fna --gff_in mygenome.gff --outdir mygenome.prophet*
5. *fastANI --rl ANI.txt --ql ANI.txt -o output.tx*
6. *~/get_homologues-x86_64-20210828/compare_clusters.pl -d gbk_homologues/genome_f0_0taxa_algOMCL_e1_S75_ -o 24.pan.e1.intersection -n -t 0 -m -T; ~/get_homologues-x86_64-20210828/compare_clusters.pl -d gbk_homologues/genome_f0_0taxa_algOMCL_e1_S75_ -o 24.core.genes -n -t 24*
7. *HYPHYMPI busted --alignment genome.fa --tree neighbor-joining >busted/genome.busted*
